# Rapid and long‐lasting efficacy of high‐dose ambroxol therapy for neuronopathic Gaucher disease: A case report and literature review

**DOI:** 10.1002/mgg3.2427

**Published:** 2024-03-30

**Authors:** Kanako Higashi, Yuri Sonoda, Noriyuki Kaku, Fumihiko Fujii, Fumiya Yamashita, Sooyoung Lee, Vlad Tocan, Go Ebihara, Wakato Matsuoka, Kenichi Tetsuhara, Motoshi Sonoda, Pin Fee Chong, Yuichi Mushimoto, Kanako Kojima‐Ishii, Masataka Ishimura, Yuhki Koga, Atsuhisa Fukuta, Nana Akagi Tsuchihashi, Yoshikazu Kikuchi, Takahito Karashima, Takaaki Sawada, Taeko Hotta, Makoto Yoshimitsu, Hideyuki Terazono, Tatsuro Tajiri, Takashi Nakagawa, Yasunari Sakai, Kimitoshi Nakamura, Shouichi Ohga

**Affiliations:** ^1^ Department of Pediatrics, Graduate School of Medical Sciences Kyushu University Fukuoka Japan; ^2^ Emergency and Critical Care Center Kyushu University Hospital Fukuoka Japan; ^3^ Department of Pediatrics National Hospital Organization Fukuoka Higashi Medical Center Koga Japan; ^4^ Department of Pediatric Surgery, Graduate School of Medical Sciences Kyushu University Fukuoka Japan; ^5^ Department of Otorhinolaryngology, Graduate School of Medical Sciences Kyushu University Fukuoka Japan; ^6^ Department of Clinical Chemistry and Laboratory of Medicine Kyushu University Hospital Fukuoka Japan; ^7^ Department of Pediatrics, Graduate School of Medical Sciences Kumamoto University Kumamoto Japan; ^8^ Department of Hematology and Rheumatology, Graduate School of Medical and Dental Sciences Kagoshima University Kagoshima Japan; ^9^ Department of Clinical Pharmacy and Pharmacology Kagoshima University Graduate School of Medical and Dental Sciences Kagoshima Japan

**Keywords:** ambroxol, chaperone, Gaucher disease, genotype, glucocerebrosidase, therapy

## Abstract

Gaucher disease (GD) is a lysosomal storage disorder caused by a deficiency in the *GBA1*‐encoded enzyme, β‐glucocerebrosidase*.* Enzyme replacement therapy is ineffective for neuronopathic Gaucher disease (nGD). High‐dose ambroxol has been administered as an alternative treatment for a group of patients with nGD. However, little is known about the clinical indication and the long‐term outcome of patients after ambroxol therapy. We herein report a case of a female patient who presented with a progressive disease of GD type 2 from 11 months of age and had the pathogenic variants of p.L483P (formerly defined as p.L444P) and p.R502H (p.R463H) in *GBA1*. A combined treatment of imiglucerase with ambroxol started improving the patient's motor activity in 1 week, while it kept the long‐lasting effect of preventing the deteriorating phenotype for 30 months. A literature review identified 40 patients with nGD, who had received high‐dose ambroxol therapy. More than 65% of these patients favorably responded to the molecular chaperone therapy, irrespective of p.L483P homozygous, heterozygous or the other genotypes. These results highlight the long‐lasting effect of ambroxol‐based chaperone therapy for patients with an expanding spectrum of mutations in *GBA1*.

## Graphical abstract

We herein report a case of a 15‐month‐old girl with neurological Gaucher disease (nGD) who had a unique combination of mutant alleles (p.L483P and p.R502H) among previously reported cases with nGD. The results of the literature review of 40 patients with nGD, including the present case, highlight the rapid and long‐lasting efficacy of ambroxol‐based chaperone therapy for nGD patients with an expanding spectrum of mutations in GBA1.

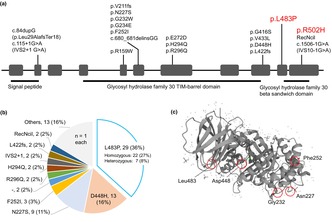

## 1 INTRODUCTION

Gaucher disease (GD) is an autosomal recessive lysosomal storage disease caused by pathogenic variations in the GBA1 gene (Suner & Delhommeau, 2022). Resulting deficits in the activity of glucocerebrosidase (GCase) lead to the accumulation of the substrates, glucocerebroside and glucosylsphingosine in tissue macrophages (Suner & Delhommeau, 2022). Affected individuals present with hepatosplenomegaly, skeletal deformity and hematopoietic cell dysfunctions. Patients without neurological symptoms receive the diagnosis of type 1 GD (GD1), and this type of disease is more frequently found in Caucasian than in Asian ancestries (Tajima et al., 2009).

GD type 2 (GD2) and type 3 (GD3) are collectively designated as neuronopathic GD (nGD) (Tajima et al., 2009) that predominantly affects the central nervous system (CNS) of patients (Narita et al., 2016). The clinical expressions of GD2 develop in infants and young children and lead to a rapid progression, distinctively from those of GD3 as the later‐onset and chronic form of the disease (Narita et al., 2016). In contrast to the dominant prevalence rate of GD1 in Western countries, 60% of patients in Asian countries are classified into those with nGD (Tajima et al., 2009). Enzyme replacement therapy (ERT; Charrow & Scott, 2015; Suner & Delhommeau, 2022) and substrate reduction therapy (Cox et al., 2015; Lukina et al., 2014; Mistry et al., 2015) are promising procedures to ameliorate the systemic symptoms in patients with GD1. However, these treatment effects are known to be limited in patients with nGD. The reason is primarily related to the fact that neither recombinant GCase nor glucosylceramide synthase inhibitors penetrate the blood–brain barrier (BBB) efficiently (Ivanova et al., 2021).

A chemical screening has identified ambroxol to be a molecular chaperone that rescues the hydrolyzing activity of mutant GCase (Maegawa et al., 2009). Because ambroxol is considered to pass the BBB, it may serve as an alternative medicine for patients with nGD. It remains to be investigated, however, whether the chaperone therapy might be also effective for patients carrying other mutations in GBA1 (Ivanova et al., 2021). In this report, we present a pediatric patient with GD2 who showed a favorable neurodevelopmental response to the high‐dose ambroxol administration with ERT during follow‐up for more than 2 years (30 months).

## 2 CASE REPORT

A 15‐month‐old girl was transferred to our hospital by ambulance for acutely exacerbating stridor and cyanosis of unidentified causes. The patient was born to unrelated, healthy parents after an uneventful pregnancy and delivery. The optional newborn screening for lysosomal diseases was not performed at the birthplace in Japan. She was noticed to have bilateral esotropia in her early infancy and was referred to an ophthalmologist at her 4‐month health check‐up. Recurrent dysphagia and cyanosis started from the age of 11 months. Hypotonia, growth arrest, and psychomotor retardation were noticed and treated at a previous hospital from the age 12 months.

**FIGURE 1:**
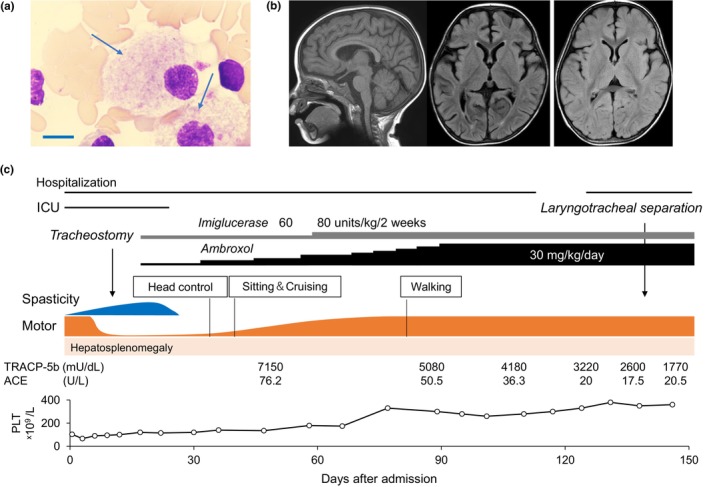
Clinical profiles and the treatment course of the present case. (a) May‐Grunwald‐Giemsa staining for Gaucher cells (arrows) in the bone‐marrow smear. Scale, 10 μm. (b) Sagittal T1‐weighted (left) and axial fluid‐attenuated inversion recovery (FLAIR, middle) image on day 24 of admission. No parenchymal lesion was detected in the FLAIR image at 3 months after discharge (right). (c) Clinical course of the patient. Treatments are shown at the top. Neurological findings and laboratory data are depicted in the middle and lower schemes, respectively. ACE, angiotensin‐converting enzyme; TRACP‐5b, Tartrate‐Resistant Acid Phosphatase 5b.

On admission, a bronchoscopic examination revealed laryngeal spasms, which required endotracheal intubation under intensive care. She had bilateral ptosis, esotropia and marked hepatosplenomegaly; liver and spleen were palpable 5 cm below the costal margins, respectively. Blood gas analysis excluded metabolic acidosis. Blood tests showed thrombocytopenia (103 × 10^9^/L) and a marginally elevated level of aspartate transaminase (AST, 58 IU/L). NH_3_ (63 μg/mL), alanine aminotransferase (ALT, 18/μL) and creatine kinase (CK, 142 U/L) levels were unremarkable. Serum angiotensin‐I converting enzyme (ACE) and acid phosphatase levels were increased to 76.2 IU/L (reference range [rr]: 8.3–21.4) and 7150 (rr: 120–420), respectively. Echocardiography showed no abnormality. A bone marrow aspiration identified Gaucher cells (Figure 1a). Within a few days after admission, opisthotonic postures recurrently emerged with or without sensory stimulations. Repeated cranial MRI determined no degenerative lesions in the CNS (Figure 1b).

**FIGURE 2:**
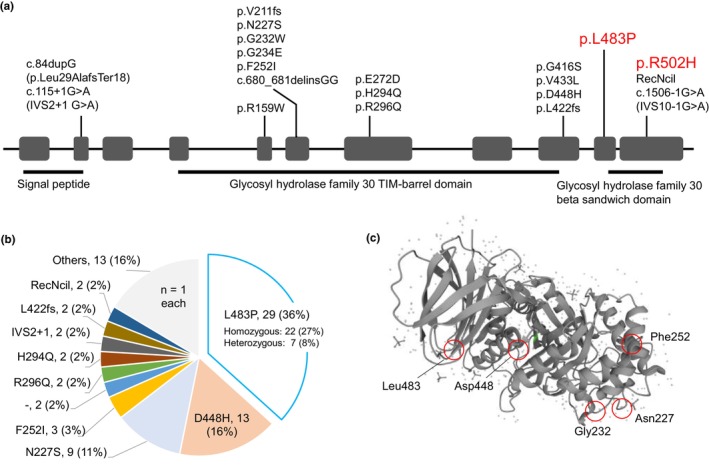
A mutational landscape of 40 patients who received ambroxol therapy for neuronopathic Gaucher disease. (a) The gene structure of *GBA1* located at chromosome 1q22. Shaded boxes represent the 11 protein‐coding exons spanning the 6.8‐kb region in the negative strand of the human genome. The left‐to‐right direction corresponds to that of transcription. Annotations indicate 23 pathogenic variants identified in patients with neuronopathic Gaucher disease. Horizontal bars represent the domain structure of GCase. Red: mutant alleles identified in the present patient. All the 40 patients having these variants were reported to receive the ambroxol treatment. (b) Pie chart indicates the number and frequency (%) of mutant alleles among 80 alleles identified in the 40 patients (panel a). —, unidentified variant. (c) A stereographic structure of GCase (UniProt: A0A068F658). The location of amino acid residues harboring nGD‐associated variants is schematically shown.

In total, 18 days after admission, the GCase activity was reported to decrease to 1.05 pmol/h/disk (reference range: 4.1–9.7). During the turn‐around time to the genetic diagnosis, we started dripping infusions of imiglucerase (60 units/kg every other week) and oral administration of ambroxol (3 mg/kg/day) from day 20 after admission. The dose of ambroxol was increased by 3 mg/kg every 2–4 weeks to reach the target doses (30 mg/kg/day; Figure 1c; Narita et al., 2016). Sanger sequencing confirmed that the patient had the compound heterozygous variants of NM_001005741.2(GBA1): c.1448T>C (p.L483P) and c.1505G>A (p.R502H) (Figure 2a, Table S1, Figure S1). These variants were previously designated as p.L444P and p.R463H, respectively. According to the InterVar database (https://wintervar.wglab.org/), the former variant (p.L483P: rs421016) has been reported to be “pathogenic”, and the latter (p.R502H: rs80356772) is defined as “uncertain significance”. Polyphen‐2 (http://genetics.bwh.harvard.edu/pph2/index.shtml) predicted the amino acid substitution of p.R502H as “probably damaging” (score: 0.999) and Mutation Taster (https://www.mutationtaster.org/) showed “disease causing” (probability >0.999), whereas SIFT (https://sift.bii.a‐star.edu.sg/index.html) supported the result of “tolerated” (score: 0.06). We further classified p.R502H as “likely pathogenic” based on ACMG (American College of Medical Genetics and Genomics) criteria (Richards et al., 2015; criteria applied: PS3 + PM3_Strong+ PM2_Supporting + PP4).

After the start of imiglucerase and ambroxol therapy, the patient began to roll over in 1 week and to sit unaided in 2 weeks (Figure 1c). During this period, spasticity and opisthotonus disappeared. The platelet counts increased to 150–200 × 10^9^/L within 2 months of treatment. Because prolonged dysphagia and laryngospasm hampered to achieve a safe and successful extubation, tracheostomy was performed on day 9 after admission. She was discharged from our hospital on day 48 after admission. Given the chronic dyspepsia, the dose of imiglucerase was increased to 80 units/kg every other week from age 19 months. She then underwent laryngotracheal separation to control recurrent airway infections at the age of 27 months. Esophageal hiatal hernia was surgically repaired at age 3 years and 6 months.

Serial monitoring of molecular markers supported the efficacy of the treatments. Increased ACE levels declined from 76.2 U/L on admission to 50.5 U/L in 1 month and to 20.5 U/L at the latest evaluation (age 2 years and 6 months; Figure 1c). Similarly, tartrate‐resistant acid phosphatase 5b (TRACP‐5b) was initially as high as 7150 mU/dL (120–420) but declined to 1770 mU/dL during the treatment course. In addition, serum glucosylsphingosine (Lyso‐Gb1) showed a decreasing trend from 720.7 ng/mL (health control; 3.8) at the beginning of treatment to 129.7 ng/mL at 5 months after the start of treatment. No serious adverse events were observed during the follow‐up period.

At the present age of 3 years 9 months, the patient walks independently, and her cognitive function has reached 12–16 months of developmental age. The health‐related quality of life score (PEDsQL™ 4.0 Generic Core Scales; Varni et al., 1999) showed marked improvement from before treatment (score: 29.3) to 2 years after starting ambroxol therapy (score: 76.0), reflecting a good quality of life with treatment.

## 3 REVIEW OF THE LITERATURE

Through the literature search from 2016 to 2022, we found a total of 39 patients with nGD who received high‐dose ambroxol therapy (Figure 2b, Table S1) (Charkhand et al., 2019; Chu et al., 2020; Ciana et al., 2020; Darling et al., 2021; Istaiti et al., 2021; Kim et al., 2020; Narita et al., 2016; Pawlinski et al., 2018; Ramadza et al., 2021). Reported patients received ambroxol therapy (25 mg/kg/day–1485 mg/day) at age 0 months to 60 years. The follow‐up period after the treatment ranged from 1 to 84 months. Among 39 patients, 18 (46.1%) had p.L483P in *GBA1* (p.L483P homozygous, *n* = 11; and heterozygous, *n* = 7), while the remaining 21 carried the other variants (Bendikov‐Bar et al., 2013; Ivanova et al., 2018) (Table 1 and Table S1). Favorable responses to ambroxol were observed in both p.L483P homozygous and heterozygous groups (>80%, Table 1). Patients without the p.L483P allele were also reported to show good responses to the treatment (66.7%). Treatment responses were hardly predictable from the positions of mutated amino acid residues in GCase (Figure 2a,c). These data suggest that the therapeutic effect of high‐dose ambroxol may not be restricted to particular genotypic subgroups of patients with nGD.

**TABLE 1:** The response rate of patients who received ambroxol therapy.

Genotype of *GBA1*	*N*	GD2 (%)^a^	Treatment response		Response rate (%)
			Yes	No (or not described)	
p.L483P, homozygous	11	0 (0)	9	1 (1)	≥81.8
p.L483P, heterozygous	7	4 (50)	6	0 (1)	≥87.5
Others (no p.L483P allele)	21	4 (19)	14	1 (6)	≥66.7

^a^ N of patients with GD2 (Gaucher disease type 2) and the percentage in each group.

## 4 DISCUSSION

We have first demonstrated the response of a young girl with GD2 to more than 30 months of ambroxol and imiglucerase therapy. This patient showed a favorable neurological development without adverse events during the follow‐up period. The literature review of 40 patients including the present one determined >65% of response rate to ambroxol and ERT irrespective of the genotype of nGD. Considering both the neuroprotective and less deleterious effects of ambroxol, the treatment protocol might be worth proposing for broader populations of patients with nGD.

Five Japanese patients with nGD have been shown to favorably respond to high‐dose ambroxol (Narita et al., 2016). In these patients, the chaperone therapy improved myoclonic seizures and ocular apraxia. Consistently, their laboratory data showed increased lymphocyte GCase activity and decreased glucosylsphingosine levels in the cerebrospinal fluid (Narita et al., 2016). In agreement with this perspective, rapid escalation in the administration dose of ambroxol is known to produce faster and better outcomes (Kim et al., 2020). More recently, a longitudinal study showed that ambroxol was safely and effectively used for siblings with GD3 (one from age 5 to 12 years; the other from age 7 weeks to 7 years; Ramadza et al., 2021). Thus, in the era of expanding newborn screening, ambroxol will serve as a preemptive therapy for infants at risk of developing nGD.

Ambroxol acts as a molecular chaperone that improves the decreased activity of mutant GCase (Maegawa et al., 2009). Besides this well‐defined mechanism, recent studies have suggested that eliglustat and ambroxol improved autophagy‐lysosome dynamics and mitochondrial functions, thereby enhancing the GCase activity in vitro (Ivanova et al., 2021). Of note, mutations in GBA1 are associated with Parkinson disease (PD) in adults, and ambroxol has been effectively used for these patients (Istaiti et al., 2021; Mullin et al., 2020). The emerging molecular models may rationalize the clinical findings that ambroxol showed therapeutic effects on symptoms of PD without GBA1 mutations (Mullin et al., 2020). These results suggest that ambroxol may not only rescue the enzymatic activity of mutant GCase, but also recover misfolded proteins in various tissues.

As in previous reports, our patient was treated with ERT and ambroxol, and her improvement in blood data and neurological symptoms could be attributed to the combination of these agents. On the other hand, the clinical course of our patient suggests that ambroxol does not necessarily improve brainstem dysfunction leading to laryngospasm and dysphagia. Based on these findings, we speculate that the brainstem might be more vulnerable to lysosomal dysfunctions than neurons in the cerebral cortex. Thus, respiratory failure, cardiac arrhythmia and other signs of dysautonomia must be observed carefully during the follow‐up period.

In conclusion, the immediate start of ambroxol was effective for a young child with the unique combination of mutant alleles (p.L483P and p.R502H) among previously reported cases with nGD. Expanding newborn screening is expected to provide further evidence for the rapid and long‐lasting efficacy of chaperone therapy for infants and young children with a variety of genotypes.

## AUTHOR CONTRIBUTIONS

Kanako Higashi and Yuri Sonoda collected data, performed formal analysis, and drafted the manuscript; Fumihiko Fujii, Takaaki Sawada and Kimitoshi Nakamura performed the genetic analysis; Takahito Karashima, Taeko Hotta, Makoto Yoshimitsu and Hideyuki Terazono performed hematological analyses; Fumiya Yamashita, Sooyoung Lee, Vlad Tocan, Go Ebihara, Wakato Matsuoka, Kenichi Tetsuhara, Pin Fee Chong, Yuichi Mushimoto, Kanako Kojima‐Ishii, Masataka Ishimura, Yuhki Koga, Atsuhisa Fukuta, Nana Akagi Tsuchihashi, Yoshikazu Kikuchi, Tatsuro Tajiri and Takashi Nakagawa managed the patient and supervised data analysis; Noriyuki Kaku, Yasunari Sakai and Shouichi Ohga conceptualized the study, curated data, and edited the final manuscript. All the authors have critically reviewed and approved the final manuscript as submitted.

## FUNDING INFORMATION

The work was supported in part by Japan Society for the Promotion of Sciences (JSPS) KAKENHI Grant numbers JP22K07893 (Sonoda) and JP23K07334 (Sakai).

## CONFLICT OF INTEREST STATEMENT

The authors declare that there is no conflict of interest concerning this work.

## ETHICS STATEMENT

This study was conducted in compliance with the institutional guidelines for clinical research. The specific protocol for retrospective analysis was approved by the Institutional Review Board at Kyushu University (#2021‐125 and #461‐04).

## CONSENT

Written informed consent was obtained from the parents.

## Supporting information


Table S1.
Figure S1.

## Data Availability

The data that support the findings of this study are available on request from the corresponding author. The data are not publicly available due to privacy or ethical restrictions.
