# Supplementary material for: Rapid and long‐lasting efficacy of high‐dose ambroxol therapy for neuronopathic Gaucher disease: A case report and literature review
Source: Mol Genet Genomic Med. 2024 Mar 30;12(4):e2427. doi: 10.1002/mgg3.2427 (PMC10980885; doi:10.1002/mgg3.2427)

**Table S1.** Summary of 40 patients who received ambroxol treatment for neuronopathic Gaucher disease

| ID | Age^a^ | Sex | GD | *GBA* genotype^b^ | Duration | |  | ABX treatment | | |  | Additional Information | Reference |
| --- | --- | --- | --- | --- | --- | --- | --- | --- | --- | --- | --- | --- | --- |
|  |  |  |  |  | ERT  (years) | ABX  (months) |  | Max dose (mg/day) | Adverse events | Discontinuation | Efficacy |  |  |
| 1 | 1y6m | F | 2 | L483P/c.680_  681delinsGG (L444P/c.680_ 681delinsGG) |  | 5 |  | 180 | Died | Yes |  |  | Istaiti, 2021 |
| 2 | 2y | F | 2 | L483P/c.115+1  (L444P/IVS2+1) | 2 | 24 |  | 380 |  |  | Improved |  | Istaiti, 2021 |
| 3 | 2y | M | 2 | L29fs/V433L  (84GG/V394L) | 2 | 1 |  | 150 | Mucus,  Cough | Yes |  |  | Istaiti, 2021 |
| 4 | 3y | F | 2 | F252I/RecNcil^c^  (F213I/RecNcil) | 2 | 24 |  | 375 |  |  | Improved |  | Narita, 2016 |
| 5 | 0y8m | F | 2/3 | L483P/RecNcil  (L444P/RecNcil) | 7 | 10 |  | 30 mg/kg |  |  | Improved |  | Chu, 2019 |
| 6 | 5y | M | 2/3 | D448H/L422fs  (D409H/L422fs) | 3 | 1 |  |  | Tremor | Yes | Improved | Add-on: Levodopa | Darling, 2021 |
| 7 | 5y | F | 2/3 | D448H/L422fs  (D409H/L422fs) | 3 | 1 |  |  |  | Yes | Improved | Add-on: Levodopa | Darling, 2021 |
| 8 | 0y7w | M | 3 | H294Q/D448H  (H255Q/D409H) | 0 | 84 |  | 25 mg/kg | Mucus, Loose stool |  | Improved |  | Ramadza, 2021 |
| 9 | 3y | F | 3 | D448H/D448H  (D409H/D409H) | 3 | 19 |  | 200 |  |  |  |  | Istaiti, 2021 |
| 10 | 4y | M | 3 | L483P/L483P  (L444P/L444P) | 3 | 1 |  | 150 | Cough | Yes |  |  | Istaiti, 2021 |
| 11 | 5y | F | 3 | D448H/D448H  (D409H/D409H) | 4 | 48 |  | 360 |  |  |  |  | Istaiti, 2021 |
| 12 | 5y | F | 3 | L483P/L483P  (L444P/L444P) | 4 | 17 |  | 250 |  |  | Prevented deterioration |  | Istaiti, 2021 |
| 13 | 5y | M | 3 | L483P/L483P  (L444P/L444P) | 4 | 20 |  | 388 |  |  | Prevented deterioration |  | Istaiti, 2021 |
| 14 | 5y | F | 3 | L483P/L483P  (L444P/L444P) | 3 | 16 |  | 248 |  |  | Prevented deterioration |  | Istaiti, 2021 |
| 15 | 5y | F | 3 | H294Q/D448H  (H255Q/D409H) | 0.2 | 84 |  | 25 mg/kg | Mucus,  Loose stool |  | Improved |  | Ramadza, 2021 |
| 16 | 6y | M | 3 | L483P/L483P  (L444P/L444P) | 5 | 20 |  | 435 |  |  | Prevented deterioration |  | Istaiti, 2021 |
| 17 | 7y | M | 3 | N227S/R159W  (N188S/R120W) | 5 | 55 |  | 420 |  |  | Improved |  | Istaiti, 2021 |
| 18 | 7y | F | 3 | L483P/L483P  (L444P/L444P) | 6 | 48 |  | 660 |  |  | Improved |  | Istaiti, 2021 |
| 19 | 7y | F | 3 | L483P/F252I  (L444P/F213I) | 4 | 13 |  | 373 |  |  | Prevented deterioration |  | Istaiti, 2021 |
| 20 | 12y | F | 3 | H294Q/L483P  (H255Q/L444P) | 7 | 49 |  | 1275 |  |  | Prevented deterioration |  | Istaiti, 2021 |
| 21 | 12y | M | 3 | L483P/L483P  (L444P/L444P) | 10 | 19 |  | 1200 |  |  | Improved |  | Istaiti, 2021 |
| 22 | 12y | F | 3 | L483P/L483P  (L444P/L444P) | 8 | 12 |  | 150 |  |  | Prevented deterioration |  | Istaiti, 2021 |
| 23 | 14y | F | 3 | N227S/c.115+1  (N188S/IVS2+1) | 8.5 | 42 |  | 25 mg/kg |  |  | Improved |  | Ciana, 2020 |
| 24 | 15y | F | 3 | N227S/V211fs  (N188S/ c.630delC) | 5 | 76 |  | 1377 |  |  | Improved |  | Istaiti, 2021  Kim, 2020 |
| 25 | 15y | F | 3 | N227S/NA  (N188S/NA) | 0.3 | 36 |  | 1300 |  |  | Improved |  | Narita, 2016 |
| 26 | 16y | M | 3 | G416S/G234E  (G377S/G195E) | 14 | 36 |  | 1300 |  | Yes | Ineffective |  | Charkhand, 2019; Istaiti, 2021 |
| 27 | 17y | F | 3 | D448H/D448H  (D409H/D409H) | 15 | 10 |  | 600 |  | Yes |  |  | Istaiti, 2021 |
| 28 | 18y | F | 3 | N227S/R296Q  (N188S/R257Q) | 6 | 76 |  | 1215 | Mucus, Cough |  | Improved |  | Istait, 2021; Kim, 2020 |
| 29 | 18y | F | 3 | F252I/L483P  (F213I/L444P) | 15 | 76 |  | 1300 | Proteinuria |  | Improved |  | Istait, 2021; Kim, 2020 |
| 30 | 19y | M | 3 | D448H/D448H  (D409H/D409H) | 13 | 10 |  | 600 |  | Yes |  |  | Istaiti, 2021 |
| 31 | 20y | F | 3 | E272D/L483P  (E233D/L444P) | 19 | 72 |  | 1300 |  |  | Improved |  | Istaiti, 2021 |
| 32 | 20y | F | 3 | N227S/NA  (N188S/NA) | 0.3 | 36 |  | 1200 | hypouricemia |  | Improved |  | Narita, 2016 |
| 33 | 21y | F | 3 | N227S/R296Q  (N188S/R257Q) | 7 | 76 |  | 1485 |  |  | Improved |  | Istait, 2021; Kim, 2020 |
| 34 | 22y | F | 3 | N227S/R502H  (N188S/R463H) | 18 | 48 |  | 1300 |  |  |  |  | Charkhand, 2019; Istaiti, 2021 |
| 35 | 25y | F | 3 | D448H/c.1506-1  (D409H/IVS10-1) | - | 6 |  | 625 |  |  | Improved | BMT | Narita, 2016 |
| 36 | 28y | F | 3 | N227S/G232W  (N188S/G193W) | 8 | 48 |  | 1200 | Skin rash,  hypouricemia |  | Improved |  | Narita, 2016 |
| 37 | 39y | F | 3 | L483P/L483P  (L444P/L444P) | 20 | 60 |  | 300 |  |  | Improved |  | Istait, 2021; Pawlinski, 2018 |
| 38 | 39y | F | 3 | L483P/L483P  (L444P/L444P) | 14 | 48 |  | 225 |  |  | Decreased pain |  | Istait, 2021 |
| 39 | 60y | M | 3 | L483P/L483P  (L444P/L444P) | 23 | 12 |  | 1300 | Abdominal  discomfort | Yes | Ineffective |  | Ciana, 2020 |
| 40 | 1y3m | F | 2 | L483P/R502H  (L444P/R463H) | 0.2 | 2 |  | 30 mg/kg |  |  | Improved |  | This study |

^a^ Age represents years (y), months (m) and weeks (w).

^b^ GBA genotypes were updated according to the nomenclature of Human Genome Variation Society (HGVS). Parentheses indicate the genotypes in the literature.

^c^ RecNcil is the complex mutation consisting of p.L483P, p.A495P and p.V499V.

ABX, ambroxol therapy; BMT, bone marrow transplantation; ERT, enzyme replacement therapy; F, female; GD, Gaucher disease; GBA, glucocerebrosidase; M, male; NA, not available.

**Figure S1.** Electropherograms of the *GBA1* variants in this family.

The upper panel shows compound heterozygous variant of the patient, NM_001005741.2: c.1448T>C (p.L483P) and c.1505G>A (p.R502H). The lower panels present heterozygous variants of her father and mother, respectively. Arrows indicate these variants.


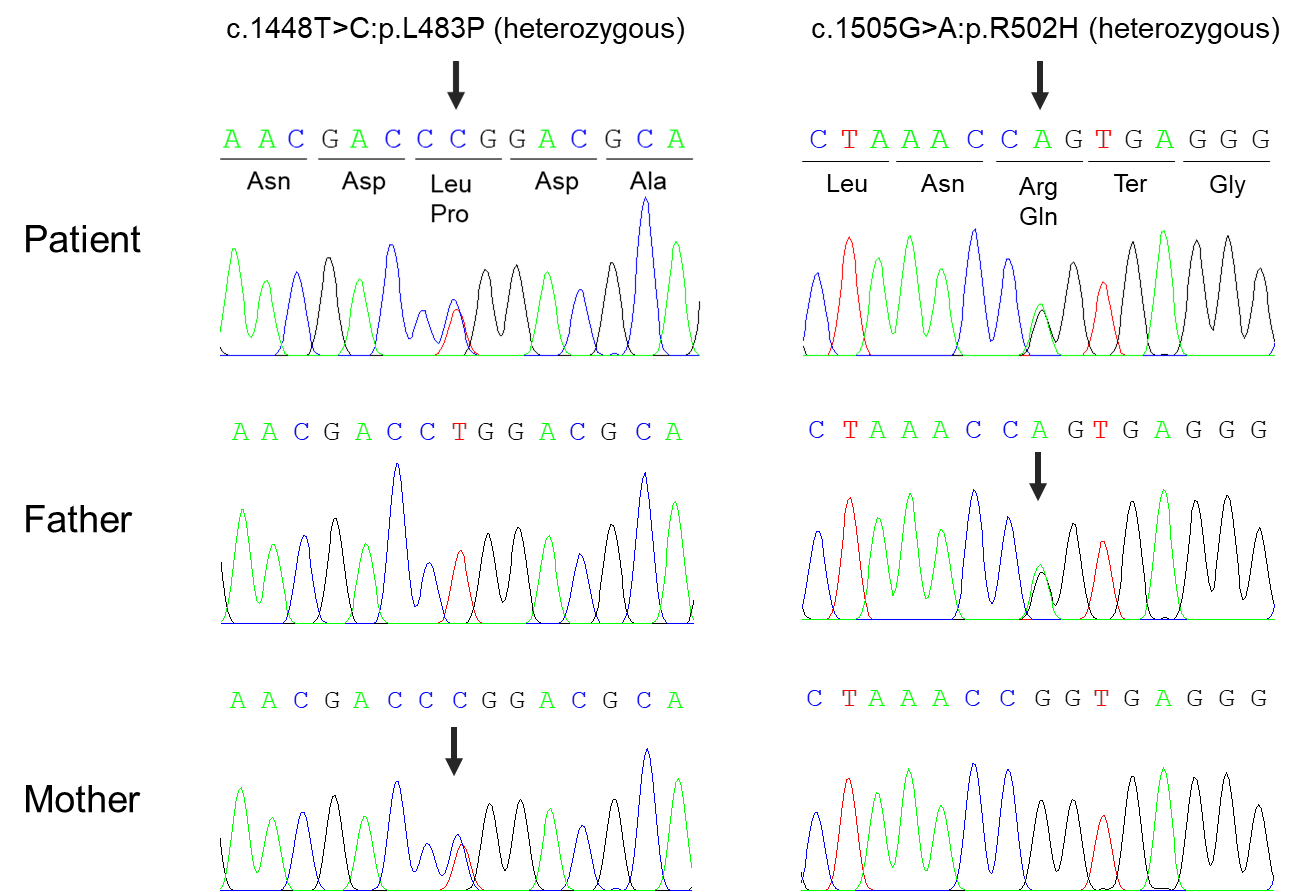

Supplement: Supplementary file 1 — Table S1. Figure S1. [file MGG3-12-e2427-s001.docx]
